# Dopaminergic Receptor Targeting in Multiple Sclerosis: Is There Therapeutic Potential?

**DOI:** 10.3390/ijms22105313

**Published:** 2021-05-18

**Authors:** Mikhail Melnikov, Mikhail Pashenkov, Alexey Boyko

**Affiliations:** 1 Department of Neurology, Neurosurgery and Medical Genetics, Pirogov Russian National Research Medical University, 117997 Moscow, Russia; boykoan13@gmail.com; 2 Department of Neuroimmunology, Federal Center of Brain Research and Neurotechnology of the Federal Medical-Biological Agency of Russia, 117997 Moscow, Russia; 3 Laboratory of Clinical Immunology, National Research Center Institute of Immunology of the Federal Medical-Biological Agency of Russia, 115522 Moscow, Russia; mvpashenkov@yandex.ru

**Keywords:** dopamine, Th17 cells, neuroinflammation, psychoneuroimmunology, multiple sclerosis

## Abstract

Dopamine is a neurotransmitter that mediates neuropsychological functions of the central nervous system (CNS). Recent studies have shown the modulatory effect of dopamine on the cells of innate and adaptive immune systems, including Th17 cells, which play a critical role in inflammatory diseases of the CNS. This article reviews the literature data on the role of dopamine in the regulation of neuroinflammation in multiple sclerosis (MS). The influence of dopaminergic receptor targeting on experimental autoimmune encephalomyelitis (EAE) and MS pathogenesis, as well as the therapeutic potential of dopaminergic drugs as add-on pathogenetic therapy of MS, is discussed.

## 1. Introduction

Multiple sclerosis (MS) is the most common demyelinating disease of the central nervous system [1]. Despite the progress made in recent years in the development of new methods of MS therapy, the treatment of MS patients remains one of the main problems of clinical neurology. The existing highly effective targeted therapy can reduce the activity of the disease [2]. At the same time, the treatment with second- or third-line disease-modifying drugs is associated with severe side effects because of the immunosuppressive impact of such therapy [3,4]. It is important to note that the ingress of the monoclonal antibodies in the CNS could be limited by the blood–brain barrier [5]. Therefore, they cannot directly affect the immune response in the CNS, while the neuroinflammation seems to proceed at least partially independently of immune processes in the periphery [6]. 

In this regard, the modulation of immune cell functions in the CNS, without affecting peripheral immune response, is considered a promising direction of pathogenetic therapy of CNS autoimmune diseases. Biogenic amines, such as serotonin, dopamine, norepinephrine, and epinephrine, are direct mediators of neuroimmune interaction. The studies in recent decades have shown that neurotransmitters regulate not only CNS functions but also have immunomodulatory effects. Cells in both the innate and adaptive immune systems have been found to express dopamine, norepinephrine, and serotonin receptors and produce biogenic amines. In addition, autocrine regulatory effects of dopamine on immune cells were demonstrated [7]. The data on the influence of dopamine, norepinephrine, and serotonin on experimental autoimmune encephalomyelitis (EAE) and MS course allow one to propose the therapeutic prospective of their receptor targeting in MS [7,8,9,10].

In this brief report, we overview the literature and own data on dopamine’s role in the regulation of neuroimmune interaction in EAE and MS. The therapeutic potential of dopaminergic receptor targeting in MS is discussed.

## 2. The Involvement of Dopamine in the Development of Clinical Symptoms in MS

Among the neurotransmitters involved in the regulation of neuroimmune interaction in MS, dopamine is one of the most well studied. Dopamine is most widely represented in the brain and modulates different CNS functions. The involvement of dopamine in depression, cognitive impairments, and fatigue in MS was shown [11,12,13,14]. In addition, dopamine has been shown to modulate the gut–brain axis, which plays an important role in the development of autoimmunity, psychiatric disorders, and neuroinflammation [15,16,17]. It is important to note that depression is one of the most common symptoms in MS and may aggravate its course, which could be explained by similar pathogenetic mechanisms of MS and depression, including an enhanced Th17 immune response [18,19]. Conversely, the clinical efficacy of “controlled stress” therapies, such as treatment with antidepressants, lifestyle modification, and coping strategies in MS, was shown [10,20,21]. 

It can be assumed that other neuropsychological symptoms of MS, such as cognitive impairment and fatigue, may also aggravate MS course [11,12,22]. Thus, Alvarenga-Filho et al. reported that plasma levels of IL-6 and TNF-α were higher in relapsing–remitting MS patients with fatigue than in the control group (relapsing–remitting MS patients without fatigue) [22]. They also found a positive association between IL-6 and TNF-α concentrations and fatigue severity. The cytokine production by anti-CD3/anti-CD28-stimulated PBMCs (IL-6, TNF-α, IFN-γ, IL-17, IL-22, and GM-CSF), as well as CD4^+^ and CD8^+^ T cells (IL-17 and IFN-γ), was higher in MS patients with fatigue [22]. The production of Th17 cell differentiation cytokines IL-6, TNF-α, IL-1β, and IL-23 by LPS-activated monocytes also was higher in MS patients with fatigue. Again, they found a correlation between the severity of fatigue and TNF-α, IL-6, IL-17, IL-22, and GM-CSF production by stimulated T cells and IL-6, IL-1β, and IL-23 production by LPS-activated monocytes [22]. At the same time, combined exercise training reduces fatigue severity, pro-inflammatory cytokine production and regulates dopamine-mediated immunomodulation of T cells in relapsing–remitting MS patients [23]. 

On the other hand, secondary dysregulation of catecholamines in MS due to neuroinflammation and structural damage of catecholamine-dependent pathways within the brain was shown [18,24,25]. In particular, Carandini et al. showed that axonal damage imbalances dopamine and norepinephrine neurotransmission in relapsing–remitting MS patients [18]. They also showed a central role of the dopamine mesocorticolimbic pathway in fatigue in MS [18]. Carotenuto et al. reported the altered functional connectivity in the dopaminergic network in MS patients compared to healthy subjects [25].

Although the concentration of biogenic amines in peripheral blood is not stable and can depend on the different factors, the change of dopamine plasma level in MS was also reported. It was shown that the plasma dopamine level in relapsing–remitting MS patients during relapse was lower than in MS patients during clinical remission or in healthy subjects [26]. Escribano et al. also showed the decreased level of dopamine in patients with MS [27].

**Figure 1:**
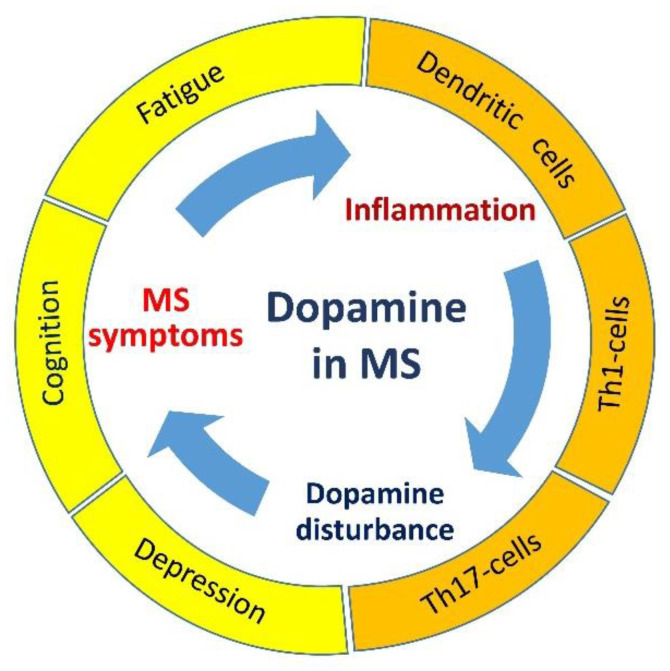
The possible role of dopamine in the development of clinical symptoms and autoimmune inflammation in MS. The dopaminergic system disturbance causes MS neuropsychological symptoms, which may aggravate MS course by the induction of pro-inflammatory cytokine production. MS exacerbation may induce a disturbance of dopamine metabolism and increase neuropsychological symptom severity.

Thus, dopaminergic system disturbance may affect MS pathogenesis by the influence on neuropsychological symptoms (development or reduction) followed by modulation of neuroinflammation and, therefore, creating one of the ‘vicious circles’ of MS (Figure 1).

## 3. The Role of Dopamine in Regulation of Neuroimmune Interaction in MS

The Th17 immune response is known to be involved in the pathogenesis of several autoimmune diseases, including MS [28]. Th17 cells produce pro-inflammatory cytokines such as interleukin (IL)-17, IL-21, IL-22, granulocyte- and granulocyte-macrophage colony-stimulating factors (G-CSF and GM-CSF). The pathogenetic role of Th17 cells in MS pathogenesis could be explained by their ability to transmigrate into CNS through the blood–brain barrier by expressing chemokine receptor 6 (CCR6 [CD196]) and producing IL-17 and IL-22 [29,30]. The presence of Th17 cells and IL-17 in the foci of EAE and MS, as well as an increase in the number of Th17 cells in blood and IL-17 production by activated peripheral blood mononuclear cells (PBMCs) in vitro, was demonstrated in MS patients during relapse compared with MS patients in clinical remission or with healthy subjects [26]. Mice knocked out by IL-17A or IL-23 have been shown to be resistant to EAE. The same effect on EAE development has IL-17 or IL-23 neutralization [31,32,33]. Finally, the role of Th17 cells on MS pathogenesis is confirmed by the influence of disease-modifying drugs on Th17 functions [34]. 

Recent studies have shown the ability of dopamine to modulate Th17 cell function. According to literature data, dopamine has various effects on Th17 cells. According to different reports, dopamine may either increase or reduce IL-17 production in MS [35]. Possibly, this could depend on the micro-environmental conditions and dopamine concentration. Depending on the concentration, dopamine may activate different dopaminergic receptors, which have different affinity and functions [35]. It is well known that there are at least five subtypes of dopamine receptors (D_1_–D_5_). D_1_- and D_5_-dopaminergic receptors (D_1_-like family) are coupled to Gαs and activate adenylate cyclase enzyme, increasing the level of cyclic adenosine monophosphate (cAMP). By contrast, D_2_-, D_3_-, and D_4_-dopaminergic receptors (D_2_-like family) are coupled to Gαi and inhibit adenylate cyclase enzyme, decreasing the cAMP level [36].

However, the involvement of dopaminergic receptors in modulation of Th17 cell function is not sufficiently investigated. According to our previous studies, the inhibitory effect of dopamine on Th17 cells could be mediated by the D_2_-like dopaminergic receptor activation. Thus, it has been shown, that D_2_-like dopaminergic receptor antagonist sulpiride abolishes dopamine-mediated IL-17 suppression in PBMC culture obtained from relapsing–remitting MS patients, while D_1_-like dopaminergic receptor antagonist SCH23390, conversely, further reduces dopamine-mediated inhibition of IL-17 production [26]. It has also been shown that SCH23390 exerts a direct inhibitory effect on IL-17, IFN-γ, and GM-CSF production by anti-CD3 and anti-CD28-activated CD4^+^ T cells in relapsing–remitting MS patients, while sulpiride has no effect on cytokine production [37]. These data correspond with data from Huang et al., who showed the inhibitory effect of D_2_-like dopaminergic receptor agonist quinpirole on the expression of Th17- and Th1-specific transcription factors (ROR-γt and T-bet, respectively) as well as IL-17, IL-22, IFN-γ, and IL-2 mRNA expression in concanavalin A-activated T-lymphocytes obtained from mesenteric lymph nodes of mice. Conversely, quinpirole increased FOXP3 and TGF-β mRNA expression, which indicates the different effect of D_2_-like dopaminergic receptor activation in pro-inflammatory Th1/Th17 cells and anti-inflammatory Treg cells [38]. In line with these data, Cosentino et al. reported the inhibitory effect of dopamine on human CD4^+^ and CD8^+^ Treg cells through D_1_-like dopaminergic receptor [39,40].

**Figure 2:**
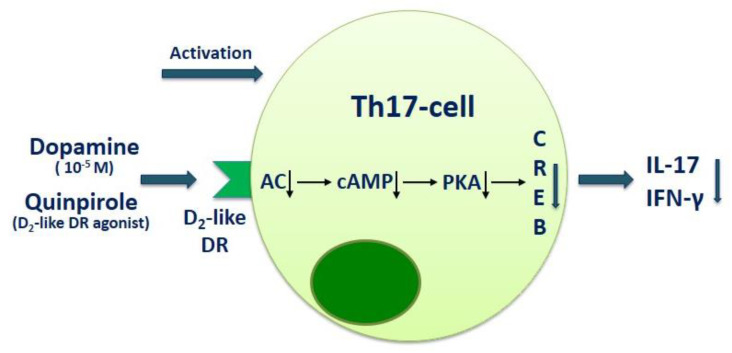
The role of dopamine and D_2_-like dopaminergic receptor in modulation of Th17 cell function. D_2_-like DR—D_2_-like dopaminergic receptor; CA—adenylyl cyclase; cAMP—cyclic adenosine monophosphate; PKA—protein kinase A; CREB—cAMP response element-binding protein.

Huang and coauthors also found that activation of D_2_-like dopaminergic receptor by quinpirole decreased intracellular cAMP content and reduced the phosphorylated cAMP-response element-binding (CREB) level in T cells. The D_2_-like dopaminergic receptor antagonist haloperidol blocked the effects of quinpirole. These data suggest that D_2_-like dopaminergic receptor modulate the cAMP-protein kinase A (PKA)-CREB pathway and regulate Th17 cell function (Figure 2) [38].

The effect of the D_2_-like receptor agonist on Th17 cells was observed also in vivo in mice with EAE. The study by Lieberknecht et al. showed that treatment of EAE mice with a pramipexole (D_2_- and D_3_-dopaminergic receptor agonist) reduced IL-17 production in lymph nodes and prevented clinical signs of the disease [41]. In this regard, D_2_-like dopaminergic receptor attracts attention as a new therapeutic target in MS. However, the molecular mechanisms that mediated D_2_-like dopaminergic receptor on Th17 cells need to be clarified.

It is important to discuss the effect of dopamine on mononuclear phagocytes, including dendritic cells and macrophages. The etiology and triggering mechanisms of the autoimmune inflammation in MS are still unclear. Dendritic cells and macrophages are professional antigen-presenting cells and play a central role in innate immune system functioning. Both types of these cells are present in the CNS. Depending on the phenotype, dendritic cells and macrophages can support autoimmune inflammation and immunological tolerance to self-antigens [42,43,44]. It is important to note that resident macrophages of the CNS form microglia are capable of producing cytokines, presenting antigens’ underlying mechanisms of neuroinflammation and MS progression [43,45]. It has been shown that in both acute and chronic foci of demyelination in MS, macrophages are more abundant to T- and B-lymphocytes [46,47,48]. 

In the demyelination lesions, macrophages can comprise cell populations: resident glial cells of the CNS and macrophages that penetrate the blood–brain barrier from the periphery to the CNS (infiltrating macrophages). According to literature data, the activation of microglial cells mediates the early neuroinflammation stage [49]. Conversely, infiltrating macrophages mediate the effector phase of demyelination [50]. It is important to note that in healthy brain tissue, there are no infiltrating macrophages [43].

The influence of dopamine on dendritic cells that induce Th17 immune response has been shown. In a study by Nakano et al., it was shown that blockade of D_1_-like dopaminergic receptor by specific antagonists (SCH23390, SKF83566 or LE300) on human dendritic cells inhibited their ability to induce Th17-immune response. Conversely, blockade of D_2_-like dopaminergic receptor by L750667, sulpiride, or nemonapride (D_2_-like dopaminergic receptor antagonists) enhanced dendritic cells-mediated Th17-cell differentiation. They also found that treatment with SCH23390 had a preventive and curative effect on EAE in mice, while D_2_-like dopaminergic receptor antagonist (L750667) increased the EAE severity [51]. The results of Prado et al. and Osorio-Barrios et al. studies confirm the anti-inflammatory effect of D_1_-like receptor (D_5_-receptor) deficiency on dendritic-cell-induced Th17 immune response in EAE mice [52,53,54].

The ability of dopamine to modulate macrophages and microglia function has also been shown [55]. In particular, dopamine has been shown to inhibit the production of IL-6 and IL-1β through NF-kB pathway suppression in LPS-activated murine microglial cells. However, authors did not find any effect of agonists/antagonists of D_1_- and D_2_-like dopaminergic receptor on dopamine-mediated microglial cell suppression [56]. 

**Figure 3:**
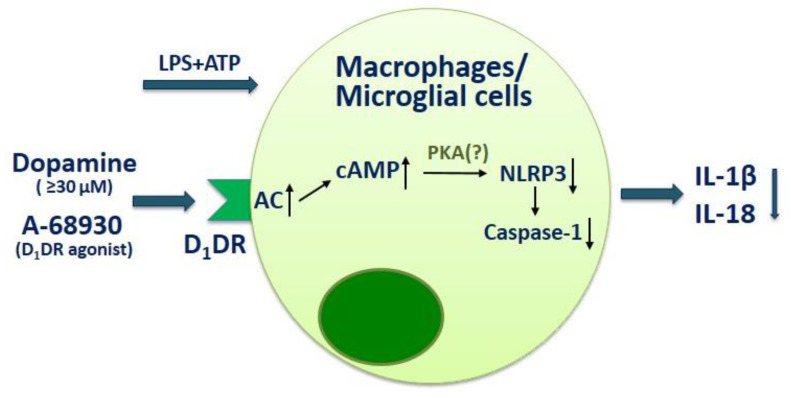
The influence of dopamine on macrophages and microglial cell function. D_1_DR—D_1_-dopaminergi receptor; CA—adenylyl cyclase; cAMP—cyclic adenosine monophosphate; PKA—protein kinase A; LPS—lipopolysaccharide; ATP—adenosintriphosphat.

Another study reported the influence of dopamine on macrophages through NLRP3-inflammasome modulation. The treatment of macrophages with dopamine reduced NLRP3-dependent caspase-1 activation as well as IL-1β and IL-18 production by microglial cells upon stimulation with LPS and adenosine triphosphate (ATP). The D_1_-dopaminergic receptor also has been shown to be involved in dopamine-mediated NLRP3 inflammasome inhibition. Thus, knockdown of D_1_-dopaminergic receptor in macrophages significantly blunted the inhibitory effect of dopamine on inflammasome activation, while agonist of D_1_-dopaminergic receptor A-68930 had the same effect with dopamine on macrophages (Figure 3) [57]. These data suggest the dual role of D_1_-like dopaminergic receptor in the modulation of the innate immunity.

In addition, the influence of atypical antipsychotic agents (such as quetiapine, risperidone, and clozapine) on EAE course through modulation of macrophages/microglia also has been shown [58,59,60]. However, these therapeutic agents affect not only dopaminergic receptors.

Taken together, these data suggest the critical role of the dopaminergic system in the control of neuroinflammatory processes. It can be proposed that dopamine may affect EAE and MS pathogenesis by mechanisms that are not mutually exclusive: by influencing neuroinflammation through the regulation of the severity of neuropsychological symptoms or by direct impact on the cells of both adaptive and innate immune systems, including resident immune cells of CNS. 

## 4. The Prospects of Dopaminergic Therapeutics in MS Treatment

The in vitro anti-inflammatory effect of dopaminergic therapeutics has been confirmed in vivo. The data on the influence of dopamine/dopaminergic receptor targeting on EAE/MS pathogenesis and course are presented in detail in Table 1.

In this regard, the repurposing of dopaminergic drugs is one of the most promising directions in the development of new therapeutic approaches in MS [8,9,35]. However, the existing data were obtained primarily in in vitro studies or EAE (an animal model of MS), while the effect of dopaminergic system targeting on MS is not sufficiently studied. In one pilot study (Bissay et al. [63]), the influence of D_2_-like dopaminergic receptor agonist bromocriptine on relapsing–remitting and progressive MS courses was tested. However, no clinical benefits of such therapy were observed [63]. It should be noted that patients who participated in these studies had not been treated with disease-modifying drugs [63]. It can be proposed that dopaminergic therapy may be more effective as an add-on to first-line disease-modifying drugs (interferon-β or glatiramer acetate). In line with this suggestion, Green et al. reported that a combination of clozapine and glatiramer acetate therapy was more effective in EAE treatment than using clozapine or glatiramer acetate alone [60]. 

Secondly, in Bissay’s study, patients with progressive MS were included. However, the MS progression is mediated by neurodegeneration, partly independent of autoimmune inflammation [65], while dopamine presumably affects inflammatory processes. Finally, this study contains just fifteen MS patients. Thus, the effect of bromocriptine on MS course needs to be clarified.

## 5. Conclusions

Taken together, the results of more than twenty years of studies suggest the potential prospect of dopaminergic therapeutics for the treatment of CNS demyelinating diseases. However, confirmation of the clinical efficacy of such therapeutics still needs to be clarified. Several reasons necessitate the development of such a therapeutic. About 30 percent of MS patients do not respond to first-line disease-modifying drugs, so it is required to switch over to more effective therapeutics [66]. At the same time, the treatment with the second- or third-line therapy may cause severe side effects. In addition, the cost of such treatment may be high. It is possible to propose that using dopaminergic therapeutics as add-on pathogenetic MS treatment will control disease course without treatment escalation. However, efficacy of such therapeutics needs to be confirmed in clinical trials.

## Figures and Tables

**Table 1:** The effect of dopaminergic receptor targeting on EAE/MS pathogenesis and course.

Disease	Cell Type	The Effect of Dopaminergic Receptor Targeting	Authors
EAE	Splenic lymphocytes	D_2_-like dopaminergic receptor agonist bromocriptine has a preventive and curative effect on EAE in mice. The treatment with bromocriptine reduces prolactin serum level and splenic lymphocyte proliferation upon Con A stimulation.	Riskind et al., 1991 [61]
EAE	Not investigated	The treatment with D_2_-like dopaminergic receptor agonist bromocriptine reduces prolactin plasma level and clinical symptoms of acute and chronic EAE.	Dijkstra et al., 1993 [62]
EAE	Dendritic cells T cells	D_1_-like dopaminergic receptor antagonist (SCH23390) has a preventive and curative effect on EAE in mice. D_2_-like dopaminergic receptor antagonist (L750667) enhances EAE severity. The spleen cells from SCH23390-treated mice produce less IL-17 than the PBS-treated mice. Dendritic cells treated with SCH23390 and transferred to mice have the same effect compared with the direct influence of SCH23390 on EAE.	Nakano et al., 2008 [51]
EAE	Dendritic cells	D_5_-dopaminergic receptor deficiency on dendritic cells impair LPS-induced IL-12 production and IL-23 mRNA expression and attenuate CD4^+^ T-cell activation. D_5_-dopaminergic receptor deficient mice show a delayed onset of the EAE and reduced disease severity compared with WT mice. Transfer of D_5_-dopaminergic receptor deficiency dendritic cells to EAE mice lessens the infiltration of Th17 cells in the CNS.	Prado et al., 2012 [52]
EAE	Peripheral lymphoid tissue	The treatment with a D_2_- and D_3_-dopaminergic receptors agonist pramipexole reduces IL-17, IL-1β and TNF-α production in lymph nodes and prevents clinical signs of the EAE in mice.	Lieberknecht et al., 2017 [41]
EAE	Dendritic cells	Transfer of D_5_-dopaminergic-receptor-deficient dendritic cells to EAE mice reduces EAE manifestation and decreases the infiltration of IL-17^+^, IFN-γ^+^IL-17^+^ and GM-CSF^+^IFN-γ^+^IL-17^+^CD4^+^ T cells at the peak of the disease.	Prado et al., 2018 [53]
EAE	CD4^+^ T cells	D_5_-dopaminergic receptor signaling in naive CD4^+^ T cells potentiates T cell activation with the acquisition of Th17-phenotype favoring EAE development. D_5_-dopaminergic receptor signaling in Treg cells contributes to their suppressive activity.	Osorio-Barrios et al., 2018 [54]
RRMS Progressive MS	Not investigated	No evidence of clinical efficacy of bromocriptine therapy in MS. After one year of treatment, 14 of the 15 patients showed disease progression.	Bissay et al., 1994 [63]
RRMS	PBMCs CD4^+^ T cells CD8^+^ T cells	Dopamine (at 10^−6^ M) enhances IL-17 and IL-21 but suppresses IL-10 and TGF-β production by PHA-activated PBMCs in RRMS patients and enhances IL-17 production by anti-CD3/anti-CD28-antibody-activated CD4^+^ and CD8^+^ T cells in RRMS patients.	Ferreira et al., 2014 [64]
RRMS	CD3^+^ T cells	Dopamine (at 10^−6^ M) enhances IL-6, IL-17, IL-21, and IL-22 but suppresses IL-10 production by anti-CD3/anti-CD28-antibody-activated CD3^+^ T cells in RRMS patients.	Alvarenga-Filho et al., 2016 [23]
RRMS	PBMCs	Dopamine (at 10^−5^ M) suppresses IL-17 and IFN-γ production by anti-CD3/anti-CD28 microbead-activated PBMCs in RRMS patients and healthy subjects. Blockade of D_1_-like dopaminergic receptor with SCH23390 enhances the inhibitory effect of dopamine on IL-17 production, while blockade of D_2_-like dopaminergic receptor with sulpiride conversely reduces it.	Melnikov et al., 2016 [26]
RRMS	CD4^+^ T cells	Dopamine (at 10^−5^ M) suppresses IL-17 and IFN-γ production by anti-CD3/anti-CD28 microbead-activated CD4^+^ T cells in RRMS patients and healthy subjects. Blockade of D_2_-like dopaminergic receptor with sulpiride reduces dopamine-mediated IL-17 suppression in MS patients. Blockade of D_1_-like dopaminergic receptor with SCH23390 reduces IL-17 and GM-CSF production by activated CD4^+^ T cells in MS patients and in healthy subjects.	Melnikov et al., 2020 [37]
